# Use of Implementation Mapping With Community-Based Participatory Research: Development of Implementation Strategies of a New Goal Setting and Goal Management Intervention System

**DOI:** 10.3389/fpubh.2022.834473

**Published:** 2022-05-10

**Authors:** Eunyoung Kang, Erin R. Foster

**Affiliations:** ^1^Program in Occupational Therapy, Washington University in St. Louis School of Medicine, St. Louis, MO, United States; ^2^Program in Occupational Therapy, Department of Neurology and Psychiatry, Washington University in St. Louis School of Medicine, St. Louis, MO, United States

**Keywords:** goals, action planning, implementation science, implementation mapping, community-based participatory research, rehabilitation, chronic condition, patient-centered care

## Abstract

**Aims:**

This study aims to identify implementation determinants, mechanisms of action, implementation strategies, and implementation outcome evaluation plans for a new theory-based rehabilitation goal setting and goal management intervention system, called MyGoals, using Implementation Mapping with community-based participatory research principles.

**Methods:**

We completed Implementation Mapping tasks 1 to 4 as a planning team consisting of MyGoals target implementers (occupational therapists (OTs), MyGoals intervention target clients (adults with chronic conditions), and the research team. We are currently conducting mapping task 5. These processes were guided by the Consolidated Framework for Implementation Research, social cognitive theory, the taxonomy of behavior change methods, and Proctor's implementation research framework.

**Results:**

We identified intervention-level determinants (*MyGoals' evidence strength & quality, relative advantages*) and OT-level determinants (*knowledge, awareness, skills, self-efficacy, outcome expectancy)*. We selected the MyGoals implementation outcome (*OTs will deliver MyGoals completely and competently*), outcome variables (*acceptability, appropriateness, feasibility, fidelity)*, and process outcomes. We also determined three performance objectives (e.g., *OTs will deliver all MyGoals intervention components)* and 15 change objectives (e.g., *OTs will demonstrate skills for delivering all MyGoals intervention components)*. Based on the identified outcomes, objectives, and determinants, we specified the mechanisms of change (e.g., *active learning)*. To address these determinants and achieve the implementation outcomes, we produced two tailored MyGoals implementation strategies: *MyGoals Clinician Education* and *MyGoals Clinician Audit & Feedback*. We developed evaluation plans to explore and evaluate how these two MyGoals implementation strategies perform using a mixed-methods study of OT-client dyads.

**Conclusion:**

We produced tailored implementation strategies for a rehabilitation goal setting and goal management intervention by using Implementation Mapping with community-based participatory research principles. The MyGoals implementation strategies may help OTs implement high-quality goal setting and goal management practice and thus contribute to bridging current research-practice gaps. Our findings can provide insight on how to apply implementation science in rehabilitation to improve the development and translation of evidence-based interventions to enhance health in adults with chronic conditions.

## Introduction

Goal setting and goal management is a core routine rehabilitation practice that can determine overall care planning, quality of care, and health outcomes (1–5). Evidence indicates that the implementation of theory-based, client-engaging goal setting and goal management can help clinicians build a better understanding of clients' goals, daily life performance, environment, etc., so they can provide quality person-centered rehabilitation to enhance clients' health (6, 7). Despite such evidence, theory-based, client-engaging goal setting and goal management is not well-implemented in current community-based rehabilitation (8).

Two major research-practice gaps in current goal setting and goal management include limited use of theory-based intervention components and poor client engagement throughout the intervention (8). Current practice often focuses on intervention components related to making goals and plans and does not sufficiently address the monitoring and adjustment of goals and plans (8). In addition, clients are often passive recipients of their rehabilitation goals, and clinicians express difficulties facilitating active client engagement during goal setting and goal management (9, 10). To address these research-practice gaps, it has been suggested that the development of a new practical and effective system that guides clinicians through the process of theory-based, client-engaging goal setting and goal management is needed (8, 10, 11).

To address this need, we developed a new system, called MyGoals, to guide occupational therapists (OTs) to implement comprehensive theory-based, client-engaging goal setting and goal management for adults with chronic conditions in community-based rehabilitation. We developed MyGoals using Intervention Mapping combined with community-based participatory research (CBPR) (12–15). MyGoals ultimately aims to enable clients to achieve personally meaningful rehabilitation goals by supporting OTs in providing a high-quality and person-centered goal setting and goal management intervention. To do so, MyGoals provides OTs with instructions, scripts, and materials for a sequence of six structured goal setting and goal management activities (*Education, Reflection, Find My Goals, Make My Goals, Make My Plans*, and *My Progress)* that they can directly apply in their practice without considerable modifications. To facilitate active client engagement, MyGoals guides OTs to use an empowerment-based approach that involves supporting clients to make self-determined decisions and actions (16). These two MyGoals approaches can help OTs deliver a theory-based, client-engaging goal setting and goal management intervention completely and competently.

Complex interventions like MyGoals require tailored and effective strategies to enhance their implementation (17, 18). If MyGoals cannot be implemented by OTs in practice as intended, it will not be efficacious nor effective in a real-life context. Therefore, it is recommended to explore and develop implementation strategies as a part of intervention development (17). This process can be rigorously navigated using an implementation science approach. Although it is not yet widely adopted in occupational therapy and rehabilitation, the use of implementation science has been identified by scholars in those fields as critical in facilitating the translation of evidence-based interventions into practice (12, 18, 19).

Implementation Mapping is an innovative implementation science approach that provides a set of systematic iterative tasks to guide implementation strategy development and evaluation (12). Implementation Mapping emphasizes the importance of using CBPR principles throughout the overall tasks (12). CBPR principles involve engaging and collaborating with community partners such as clients, clinicians, researchers, organizational representatives, policymakers, etc. to better understand the complex intervention context and facilitate the integration of real-world and academic knowledge, thus enhancing the likely effectiveness of interventions and their implementation strategies (14, 15). Implementation Mapping with CBPR principles or collaboration with community partners has shown benefits in other fields, but it has yet to be widely adopted in developing implementation strategies for rehabilitation interventions (12, 20, 21). Given its promising effects, Implementation Mapping may inform the development of effective MyGoals implementation strategies.

The purpose of this study was to use Implementation Mapping to identify MyGoals implementation determinants, mechanisms of action, implementation strategies, and outcome evaluation plans. The results from this study will provide insight into factors that influence the implementation of quality goal setting and goal management in community-based rehabilitation with adults with chronic conditions and how to address these factors to enhance its implementation. This study will also inform future efforts to apply implementation science and collaborate with community partners to develop and optimize rehabilitation interventions.

## Materials and Methods

### Overall Study Design

This is a mixed-methods study involving five Implementation Mapping tasks as a part of the MyGoals implementation strategy development and optimization process.

### Research Context and Planning Team Members

This paper reports the Implementation Mapping tasks that were completed as a part of the larger MyGoals development project. In the larger MyGoals development project, we established a planning team consisting of two OTs, two adults with chronic conditions, and the research team to develop MyGoals using Intervention Mapping (13) and to develop the MyGoals implementation strategy using Implementation Mapping (12).

We conducted a total of 10 virtual meetings using video-conference calls and in-person meetings at a research-based university in the Midwest, United States. The planning team members were asked to join the meetings when the mapping tasks and meeting agenda were directly applicable to them. The OT planning team members participated in all Intervention Mapping and Implementation Mapping tasks. The client members joined in all Intervention Mapping and Implementation Mapping tasks 4–5. Because our study first aimed to create and optimize MyGoals and its implementation strategy for community-based rehabilitation generally before targeting a specific site, we did not address the adoption and maintenance of MyGoals. The MyGoals Intervention Mapping process will be published elsewhere.

### Planning Team Eligibility and Recruitment

#### Occupational Therapists

Two OTs who met the following inclusion criteria participated as planning team members: (1) aged > 18 years old, (2) English speakers, (3) licensed OTs, (4) experience working in community-based rehabilitation settings with adult clients, (4) at least 1-year professional clinical experience relevant to goal setting and goal management with adults with chronic conditions. The exclusion criteria were (1) no access to the REDCap survey, e-mail, or internet and (2) <1 year of professional clinical experience relevant to goal setting and goal management with adults with chronic conditions to prevent a lack of clinical experience interfering with MyGoals' feasibility evaluation. The OTs were recruited by word of mouth.

#### Clients

Two clients who met the following inclusion criteria participated as planning team members: (1) aged > 18 years old, (2) English speakers, (3) have one or more chronic conditions. The exclusion criteria were (1) severe cognitive impairment or dementia defined as a total Montreal Cognitive Assessment (22) score <21 and (2) any other condition that may interfere with research participation (e.g., blindness). Client participants were recruited using a research participant registry and word of mouth.

### Theories, Models, and Frameworks for MyGoals Implementation Strategies

In implementation science, theories, models, and frameworks can be used to guide (1) the implementation process, (2) implementation determinant identification and strategy development, and (3) implementation outcome evaluation (23). In this study, we used Implementation Mapping (12), Consolidated Framework For Implementation Research (CFIR) (24), social cognitive theory (25), the taxonomy of behavior change methods suggested by Intervention Mapping (26), and Proctor's implementation research framework (27).

We used Implementation Mapping (12) to guide the overall process of identifying and optimizing implementation determinants, mechanisms of action, implementation strategies, and implementation outcome evaluation plans for MyGoals. Implementation Mapping provides five iterative tasks including (1) conducting the implementation needs assessment, (2) identifying implementation outcomes and the matrices of change, (3) selecting implementation strategies, (4) making implementation materials, and (5) evaluating implementation outcomes (12).

We used the CFIR (24) to identify MyGoals implementation determinants and guide implementation strategy development. The use of CFIR allowed us to explore and identify influential implementation contextual factors across domains. The CFIR includes intervention, individuals involved, inner setting, outer setting, and process domains (24). As mentioned above, because this study targeted community-based rehabilitation generally, not a specific site, we did not evaluate inner setting determinants. In addition, we used the CFIR-Expert Recommendations for Implementing Change (ERIC) Matching tool (28). The CFIR-ERIC Matching tool provides a list of recommended implementation strategies to address each CFIR-based determinant (28). Thus, the CFIR-ERIC matching tool provided us with potential sets of strategies to start with. To develop implementation change objectives and mechanisms of action, we used social cognitive theory (25) and the taxonomy of behavior change methods (26).

Lastly, we used Proctor's implementation research framework (27) to determine the MyGoals implementation outcomes. In this study, we evaluated the appropriateness, acceptability, and feasibility of MyGoals and MyGoals implementation strategies (27). We also evaluated the fidelity of MyGoals.

### Implementation Mapping Tasks

**Figure 1:**
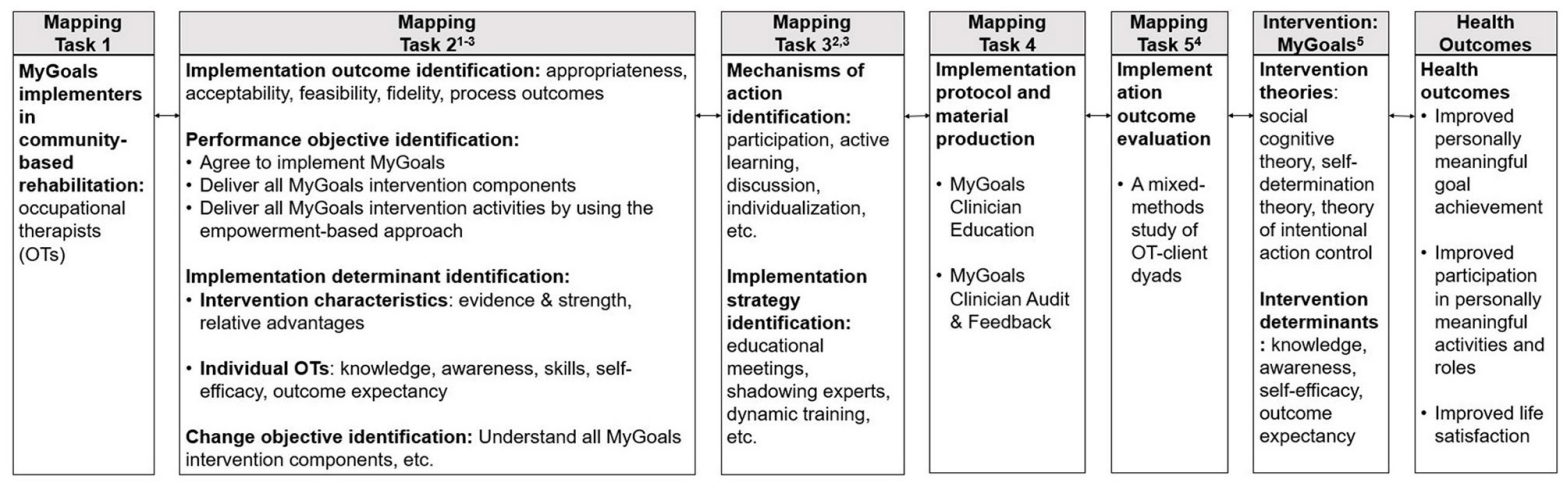
The working conceptual model for MyGoals implementation strategy development and evaluation. Guiding theories, models, and frameworks: (1) Social cognitive theory, (2) A taxonomy of behavior change methods, (3) CFIR, (4) Proctor's implementation research framework, (5) Intervention Mapping.

All Implementation Mapping tasks were completed through the planning team meetings. Throughout the meetings, we had a different agenda for each mapping task but used the same principles to maximize client and OT team members' participation in the tasks. Before the meetings, the research team prepared easy-to-understand and eye-catching meeting readings, presentations, drafts, etc. to facilitate all team members' understandings of topics and brainstorming. During the meetings, the research team reflected, summarized, and facilitated interactive discussions. The research team ensured that all members participated in discussions by explicitly asking individual members' opinions to reach a consensus for each task. After meetings, if the research team found any inconsistent content, they brought these points back and double-checked with planning team members to reach a consensus. Figure 1 describes the working conceptual model for MyGoals implementation strategy development and evaluation.

In the first task, we conducted a needs assessment through informal discussions to identify who implements MyGoals (i.e., implementers) using the following question: “Who will implement MyGoals in community-based rehabilitation settings?” In the second task, we determined implementation outcomes, performance objectives (what specific step or action MyGoals implementers need to perform to achieve the implementation outcomes), change objectives (what and how determinant needs to be changed to achieve the performance objectives), and implementation determinants. We choose all applicable implementation outcomes from Proctor's implementation research framework (27). To identify the performance objectives, we used the following question: “What do the MyGoals implementers need to do to deliver MyGoals completely and competently?” The implementation determinants were identified using the CFIR (24) and social cognitive theory (25). We used the CFIR Interview Guide Tool to determine MyGoals implementation determinants for each performance objective (29). We used all questions from the CFIR Interview Guide Tool that are designed to explore intervention, individuals involved, and process domains (29). For the outer setting domain, we only explored one determinant, *Patient Needs & Resources*, because other constructs such as *External Policies & Incentives* can vary considerably across OT inner work settings. Based on the identified determinants, we developed the change objectives and the matrices of change.

In the third task, we selected mechanisms of action and implementation strategies that are deemed applicable and effective in targeting the MyGoals implementation determinants to achieve the change and performance objectives. To choose theory- and evidence-based mechanisms of action, we first reviewed all the taxonomy of behavior change methods that are suggested effective in targeting the identified determinants and then identified ones that are applicable with the chosen implementation strategies (26). To determine the MyGoals implementation strategy, we first chose potential strategies that have shown at least 20% of experts' endorsement from the Expert Recommendations for Implementing Change (ERIC) (28) to address the MyGoals implementation determinants. We then selected and optimized final strategies that are most applicable in the current stage of MyGoals and community-based rehabilitation generally. We took into consideration the parameters for effectiveness suggested by the taxonomy of behavior change methods to translate the chosen implementation strategies more effectively and practically (26). It is important to note that the processes of identifying change methods and implementation strategies and designing these strategies based on the parameters for effectiveness were completed iteratively. As we completed these series of iterative steps to reinforce the connections among determinants, change and performance objectives, implementation strategies, and the parameters of effectiveness, we were able to design the MyGoals implementation strategies to align with the chosen determinants, the objectives, and the parameters.

In the fourth task, we produced *MyGoals Clinician Education* and *MyGoals Clinician Audit & Feedback*. We first drafted the *MyGoals Clinician Education* content. Then we optimized the *MyGoals Clinician Education* content and delivery based on the developed matrices of action and chosen implementation strategies. After the initial development of *MyGoals Clinician Education*, we conducted pilot-testing with a new OT-client dyad (identified using the same eligibility criteria and methods described above for planning team members) to optimize *MyGoals Clinician Education*. The OT completed the following tasks in order: (1) two virtual *MyGoals Clinician Education* sessions, (2) deliver MyGoals activities 1–5 to a client, (3) *MyGoals Clinician Audit & Feedback*, (4) deliver MyGoals activity 6 to the client, and (5) implementation outcome evaluations. Based on the findings from this pilot-testing, we refined *MyGoals Clinician Education, MyGoals Clinician Audit & Feedback*, and MyGoals.

In the fifth task, we specified the process evaluation question items, outcome indicators and measures, and the study design to evaluate MyGoals implementation outcomes. We are currently conducting the MyGoals implementation strategy evaluation using a mixed-methods study of OT-client dyads.

## Results

*Mapping task 1*: We identified that the MyGoals implementers are *OTs*.

*Mapping task 2*: We determined the MyGoals implementation outcome, *OTs will deliver MyGoals completely and competently*, and outcome variables including *acceptability, appropriateness*, and *feasibility* of MyGoals implementation strategies and *acceptability, appropriateness, feasibility*, and *fidelity* of MyGoals. Due to the early nature of our research, other implementation outcomes suggested by Proctor's implementation research framework (27) such as penetration, sustainability, uptake, and costs of implementation strategies were not explored in this research. We also identified three performance objectives: *(1) Agree to implement MyGoals, (2) Deliver all MyGoals intervention components*, and *(3) Deliver all MyGoals intervention activities by using the empowerment-based approach*.

We then explored MyGoals implementation determinants using all CFIR domains except the inner setting and found that intervention- and individual-level determinants are key determinants. The identified intervention-level determinants are MyGoals' *evidence & strength* and *relative advantages*. This is because MyGoals is new, so OTs are not yet aware of its evidence and benefits over other existing systems. Thus, to facilitate MyGoals implementation, it will be crucial that OTs understand its evidence and its advantages over other existing systems. The OT-level determinants are their *knowledge, awareness, skills, self-efficacy*, and *outcome expectancy*. To target these OT-level determinants, we specified change objectives for each chosen determinant. Table 1 shows the matrices of change which illustrates determinant, change objectives, and performance objectives. No outer setting- and process-level determinants were found to be critical in this research.

**Table 1:** MyGoals matrices of change.

**Performance objectives (OTs will…)**	**Change objectives (OTs will…)**				
	**Knowledge**	**Awareness**	**Skills**	**Outcome expectancy**	**Self-efficacy**
**1. Agree to implement MyGoals as intended**	1.1. Understand goal setting and goal management practice concepts and its importance 1.2. Understand evidence of MyGoals	1.3. Acknowledge that current goal setting and goal management practice is not optimal 1.4. Acknowledge that MyGoals is acceptable 1.5. Acknowledge that MyGoals is appropriate 1.6. Acknowledge that MyGoals is feasible	NA	1.7. Expect delivering MyGoals will improve personally meaningful goal achievement in clients	NA
Mechanisms of action	Participation, active learning, individualization, advance organizers, discussion, elaboration	Participation, active learning, individualization, consciousness raising, self-evaluation	NA	Participation, active learning, individualization, self-reevaluation, shifting perspective, elaboration	NA
**2. Deliver all MyGoals intervention components**	2.1. Understand all MyGoals intervention components	NA	2.2. Demonstrate skills for delivering all MyGoals intervention components completely	2.3. Expect delivering all MyGoals intervention components will improve personally meaningful goal achievement in clients	2.4. Express confidence in one's ability to deliver all MyGoals intervention components
Mechanisms of action	Participation, active learning, individualization, advance organizers, discussion, elaboration	NA	Participation, active learning, individualization, guided practice	Participation, active learning, individualization, self-reevaluation, shifting perspective, elaboration	Participation, active learning, individualization, guided practice
**3. Deliver all MyGoals intervention activities by using the empowerment-based approach**	3.1. Understand 4 MyGoals communication strategies	NA	3.2. Demonstrate skills for delivering all activities by using 4 MyGoals communication strategies	3.3. Expect using 4 MyGoals communication strategies will improve personally meaningful goal achievement in clients	3.4. Express confidence in one's ability to deliver all activities by using 4 MyGoals communication strategies
Mechanisms of action	Participation, active learning, individualization, advance organizers, discussion, elaboration	NA	Participation, active learning, individualization, guided practice	Participation, active learning, individualization, self-reevaluation, shifting perspective, elaboration	Participation, active learning, individualization, guided practice

*Mapping task 3*: Based on the identified change objectives, we selected the mechanisms of change using the taxonomy of behavior change methods (26). All selected mechanisms of change are outlined in Table 1. For a detailed description of each mechanism and parameters for effectiveness, refer to Kok et al. (26).

**Figure 2:**
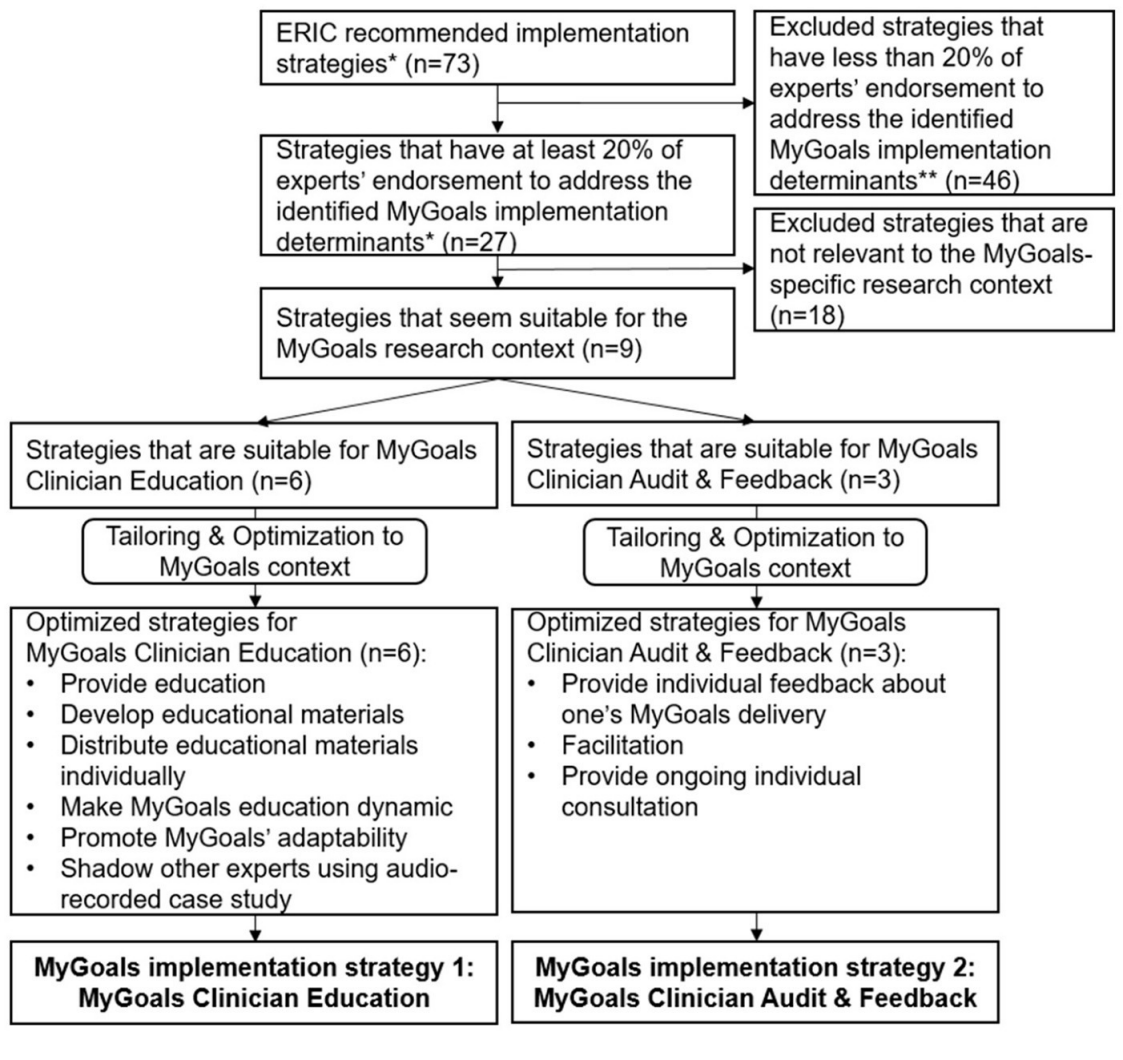
MyGoals implementation strategy selection and optimization process. *Powell et al. (28). **The identified determination determinates included MyGoals' evidence strength and quality, MyGoals' relative advantage, and OT's knowledge, awareness, skills, self-efficacy and outcome expectancy.

To develop MyGoals implementation strategies, we first selected 27 potential ERIC-recommended strategies that can address the MyGoals implementation determinants. Then we selected nine ERIC-recommended implementation strategies that can inform the development of MyGoals implementation strategies. Based on these nine strategies, we developed two MyGoals implementation strategies: *MyGoals Clinician Education* and *MyGoals Clinician Audit & Feedback*. These strategies were further enhanced by incorporating the parameters for effectiveness suggested by the taxonomy of behavior change methods (26). For instance, one of the common mechanisms of change used in this project included individualization. According to the taxonomy of behavior change methods, providing personal communication tailored to a person's needs is an essential parameter to activate the individualization change method (26). Thus, we incorporated personal communication in developing MyGoals implementation strategies by being more intentional and explicit to ask and respond to the individual OT's needs to improve the likely effectiveness of MyGoals implementation strategies. Figure 2 describes the MyGoals implementation strategy selection and optimization process.

We developed *MyGoals Clinician Education* based on the following six ERIC-recommended strategies: *conducting educational meetings, developing educational materials, distributing educational materials, making training dynamic, promoting adaptability*, and *shadowing other experts* (28). The remaining three strategies, *auditing and providing feedback, facilitation*, and *providing ongoing consultation*, were used to inform *MyGoals Clinician Audit & Feedback* (28). We described two MyGoals implementation strategies based on the reporting guideline for implementation strategies by Proctor et al. (30) in Table 2.

**Table 2:** MyGoals implementation strategies specification.

	**MyGoals Clinician Education**	**MyGoals Clinician Audit & Feedback**
Actors	The research team	The research team
Actions	• Provide MyGoals clinician education to introduce concepts, importance, and current limitations of goal setting and goal management, and MyGoals • Develop easy-to-use MyGoals instructions, script, and materials to enhance the quality of MyGoals and facilitate learning • Develop eye-catching PowerPoint for MyGoals Clinician Education to facilitate learning • Distribute MyGoals by email to provide the opportunity to thoroughly review MyGoals evidence during the self-study session • Role-play with the clinician trainee to boost confidence and perceive the potential benefits of using MyGoals • Promote MyGoals' flexible activity steps that can be tailored to each client • Provide audit and active discussion on the audio-recording of the experienced OT's MyGoals sessions to learn ideal MyGoals practice and boost one's confidence to deliver MyGoals	• Provide feedback about OT's MyGoals delivery based on direct observation of the MyGoals session to boost one's confidence for the next MyGoals delivery • Facilitate OT's reflection on areas that they performed well and areas that can be improved to reinforce the perceived benefits of using MyGoals and to support better MyGoals delivery • Provide ongoing consultation about OT's MyGoals delivery based on direct observation of the session to boost one's confidence about MyGoals delivery
Action target	Newly trained OT's knowledge, self-awareness, skills, outcome expectancy, and self-efficacy	Newly trained OT's knowledge, self-awareness, skills, outcome expectancy, and self-efficacy
Temporality	Two education sessions will be provided before any client visit	Audit & Feedback will be provided before the second visit with each client
Dose	2 sessions (2 hours each)	1 session for each client (0.5 hours)
Implementation outcomes affected	Appropriateness, acceptability, feasibility, process outcomes	Appropriateness, acceptability, feasibility, process outcomes
Justification	The six integrated ERIC recommended implementation strategies are deemed promising to address the MyGoals determinants	• The three integrated ERIC recommended implementation strategies are deemed promising to address the MyGoals determinants • Providing post-training to clinicians shows promise for enhancing the quality of intervention implementation (31)
Incorporated ERIC recommended implementation strategies (Target determinants^*^)	• Provide education (Intervention's evidence strength & quality, intervention's relative advantage, OT's knowledge) • Develop educational materials (Evidence strength & quality, OT's knowledge) • Distribute educational materials individually (Intervention's evidence strength & quality) • Make MyGoals education dynamic (OT's self-efficacy) • Promote MyGoals' adaptability (Intervention's relative advantage) • Shadow other experts using an audio-recorded case study (OT's self-efficacy)	• Provide individual feedback about one's MyGoals delivery (OT's self-efficacy) • Facilitate (Intervention's relative advantage, OT's knowledge) • Provide ongoing individual consultation (OT's self-efficacy)

* *We listed MyGoals determinants that have shown at least 20 percent of experts' endorsement from the ERIC study (28)*.

*Mapping task 4*: Based on the identified strategies and matrices of action, we drafted the *MyGoals Clinician Education* and *MyGoals Clinician Audit & Feedback* and completed pilot-testing. The results from the pilot-testing indicated that most of the developed implementation strategies seem feasible. We made minor revisions to scripts, wording, and sequence of presentation contents to streamline *MyGoals Clinician Education*. We edited the audio recordings of the experienced OT's MyGoals sessions provided as a part of *MyGoals Clinician Education* to more efficiently deliver key messages from the case examples. After the pilot-testing, we also added options for OTs to choose when and how they want to complete the *MyGoals Clinician Audit & Feedback*. In the pilot-testing, we delivered an in-person *MyGoals Clinician Audit & Feedback* right before the OT sees the client for their second visit. We found that it can be more beneficial to provide individual OTs with options for when (e.g., right after their 1st client session, between sessions, etc.) and how (e.g., virtual or in-person) they want to complete the *MyGoals Clinician Audit & Feedback*. This revision allowed us to tailor the *MyGoals Clinician Audit & Feedback* to the individual OT's learning style and preferences. We also extended *MyGoals Clinician Audit & Feedback* from 15-min to 30-min to provide enough time for OTs to discuss their feedback, concerns, questions, etc.

Table 2 describes the details of the *MyGoals Clinician Education* and *MyGoals Clinician Audit & Feedback*. The first education session aims to educate on overall goal setting and goal management concepts, practice, and application and evidence of MyGoals. The second education session aims to equip OTs to administer MyGoals with a client through role-playing with the research team member. The *MyGoals Clinician Audit & Feedback* aims to provide OTs with individualized feedback and consultation to enhance their MyGoals implementation.

*Mapping task 5*: We identified measures, respondents, and time points to evaluate the selected implementation outcomes described in Table 3. We confirmed that all selected measures worked well from the pilot testing. We will explore the preliminary effects of the MyGoals implementation strategies using quantitative measures and explore OTs' perspectives of how it may be optimized using a qualitative interview (e.g., How can we make *MyGoals Clinician Education* more feasible?).

**Table 3:** Selected outcome variables, measures, respondent, and measurement time point.

**Outcome variables**	Measures^*^
**MyGoals clinician education** & **MyGoals clinician audit & feedback**	
Acceptability	Acceptability of intervention measure (33), qualitative interview
Appropriateness	Intervention appropriateness measure (33), qualitative interview
Feasibility	Feasibility of intervention measure (33), qualitative interview
Process outcomes (Change objectives)	Quantitative questions, qualitative interview
**MyGoals**	
Acceptability	Acceptability of intervention measure (33), qualitative interview
Appropriateness	Intervention appropriateness measure (33), qualitative interview
Feasibility	Feasibility of intervention measure (33), qualitative interview
Fidelity – competence, adherence	Fidelity survey – competence and adherence scales, qualitative interview

* *All measures except fidelity will be completed by an OT after the completion of the last MyGoals session. Fidelity will be measured by both OT and observer (the research team) right after the completion of each MyGoals session*.

We also developed quantitative measures to explore how successfully the *MyGoals Clinician Education* and *MyGoals Clinician Audit & Feedback* help OTs achieve each change objective and qualitative questions to explore how to improve them. The self-report quantitative question items were developed based on the change objectives outlined in Table 1 and will be answered by using an 11-point Likert scale (0: strongly disagree −10: strongly agree). For instance, to evaluate the change objective 1.2, OTs will be asked to rate their agreement with the following item: *I understand the evidence of MyGoals*. Qualitative interview questions will be used to explore OT's perspectives on the change objectives (e.g., How can we better help you understand the evidence of MyGoals?). We are currently undergoing implementation outcome evaluation using a mixed-methods study of OT-client dyads to explore and optimize MyGoals implementation strategies in preparation for a future larger study.

## Discussion

This study aimed to develop effective strategies to ensure high-quality implementation of a goal setting and goal management intervention called MyGoals in community-based rehabilitation with adults with chronic conditions. To do so, we used Implementation Mapping with CBPR principles to determine MyGoals implementation determinants, mechanisms of action, implementation strategies, and evaluation plans. To our knowledge, this is the first study to use Implementation Mapping with CBPR principles to develop implementation strategies for a community-based rehabilitation goal setting and goal management system. We found that Implementation Mapping can guide the development and optimization of theory- and evidence-based MyGoals implementation strategies and their evaluation plans. In turn, the developed MyGoals implementation strategies may support OTs in providing better goal setting and goal management in community-based rehabilitation with adults with chronic conditions. These findings can inform future research on how to use implementation science to develop and optimize rehabilitation interventions and their implementation strategies, and thus help bridge research-practice gaps to improve health in adults with chronic conditions.

In our study, we enhanced the theoretical rigor and ecological validity of our research findings by using theories, models, and frameworks combined with CBPR principles. The collaboration and co-learning process with MyGoals implementers and MyGoals intervention target clients helped us (the research team) better understand the complex MyGoals implementation context from the end-users' perspective. If we did not actively collaborate with OT members throughout this research but merely interviewed them as research subjects, we may have been able to identify key determinants but then developed implementation strategies deemed feasible and effective from the researchers' but not clinicians' perspectives. At the same time, as much as the use of CBPR principles is important, it is critical to develop implementation strategies with theoretical rigor. To do so, we used theories, models, and frameworks as guidance to synergize the real-world and academic knowledge for developing effective MyGoals implementation strategies.

We took a holistic approach to identify determinants that will play important roles in implementing MyGoals in community-based rehabilitation. We found that having the buy-in of individual OTs can be key to facilitating MyGoals implementation. Previous literature suggests that OTs' self-awareness about their interaction with clients can promote quality goal setting practice (9). Our findings expand on this by identifying additional implementation determinants. These include OTs' skill, knowledge, self-efficacy, outcome expectancy, and MyGoals' evidence and relative advantages in the context of community-based rehabilitation. Future studies should examine if and how these determinants impact goal setting and goal management in different settings.

We identified MyGoals implementation outcome variables that can contribute to enhancing the quality of MyGoals intervention. We chose *Enabling OTs to deliver MyGoals completely and competently* as the implementation outcome. This outcome was chosen because achieving high levels of MyGoals' completeness and competency can facilitate the comprehensive use of theory-based intervention components and active client engagement. As a result, it can address the abovementioned two major research-practice gaps in community-based goal setting and goal management rehabilitation. In addition, we chose to evaluate MyGoals' and MyGoals implementation strategies' appropriateness, acceptability, feasibility, and fidelity of MyGoals. Good appropriateness, acceptability, feasibility, and fidelity are known prerequisites for high-quality intervention delivery to improve clients' health (27). Thus, we hypothesized that targeting these selected implementation outcomes will enhance MyGoals intervention quality.

We identified theory- and evidence-based mechanisms of action to facilitate MyGoals implementation and then used them to guide the MyGoals implementation strategy development. The specification of mechanisms of action is essential to understand why and how implementation strategies can enhance the implementation of interventions (32). In this study, we used social cognitive theory (25) and the taxonomy of behavior change methods (26) to clarify the mechanisms of action deemed applicable and effective for targeting the MyGoals determinants and facilitating MyGoals implementation. To produce effective implementation strategies, it is important to develop tailored strategies with clear targeted determinants and mechanisms of action (31, 32). MyGoals implementation strategies are tailored to the identified determinants and developed based on the theory- and evidence-based mechanisms of actions and the parameters of effectiveness. Given that tailored implementation strategies are known to be more effective than the non-tailored ones (31, 32), we hypothesized that MyGoals implementation strategies would be effective in achieving good appropriateness, acceptability, feasibility, fidelity, and process outcomes. Because we clearly and carefully mapped the mechanisms of action and implementation strategies, this study will advance our understanding of why and how MyGoals implementation strategies work and what aspects of these strategies require improvement to further enhance the implementation of MyGoals.

Despite existing implementation strategy reporting guidelines, many intervention studies have limited descriptions of their implementation strategies, which can hinder reliable interpretation of research findings and replication in future work (30, 32). We demonstrated that it is feasible to report implementation strategies for a rehabilitation intervention according to the guideline (30, 32). As recommended by the guideline (30), we labeled MyGoals implementation strategies consistent with the implementation science literature and defined the actors, actions, action targets, temporality, dose, target implementation outcomes, and justifications. This work will allow replication of high-quality MyGoals implementation in future studies as well as inform implementation strategies for other potential goal setting and goal management interventions. Furthermore, it may stimulate better reporting practices, and thus better synthesis and replication of future rehabilitation research in general.

Overall, we demonstrated that it is feasible to develop both MyGoals implementation strategies and MyGoals concurrently. Implementation science literature has recommended taking more active consideration of implementation strategies, ideally from the earliest stages of intervention development, to facilitate intervention translation (12). However, implementation strategies are not regularly addressed in the developmental phase of interventions in general and even more rarely in rehabilitation fields (12, 18). Our collaborative and systematic approach enabled us to develop tailored implementation strategies and enhance the adaptability of MyGoals without compromising its essential intervention components. We are currently testing MyGoals implementation strategies using a mixed-methods study of OT-client dyads based on the developed implementation outcome evaluation plans. The findings from these outcome and process evaluations will allow us to further optimize MyGoals implementation strategies and inform other works.

## Limitation

We had a comparatively small planning team. The client and OT members only had limited time to commit to this research. Both OT planning team members worked at the same university community-based clinic, so they do not represent all community-based OTs. If we could have worked with a larger number of people from different settings, from more diverse demographic and socioeconomic backgrounds, and with more protected time to work on this research throughout the study design, analysis, and manuscript writing, we could have further enhanced the overall Implementation Mapping process and produced more equitable and generalizable findings. However, to address these limitations, we incorporated multiple approaches to enable all members to actively participate in the current research study so that we were able to complete the collaborative Implementation Mapping tasks.

We endeavored to develop MyGoals implementation strategies that are deemed feasible and effective for general community-based settings, so extensive adaptation work may not be required. However, future studies may still benefit from adapting MyGoals to facilitate its implementation in specific contexts. Organizational and systematic support to allow diverse stakeholders' active and sustainable participation in research can enhance our efforts to incorporate community-engaged research in implementation science.

## Conclusion

We demonstrated that it is feasible and beneficial to develop implementation strategies using Implementation Mapping with the CBPR principles in conjunction with the development of the rehabilitation intervention itself. We identified MyGoals implementation determinants, strategies, and evaluation plans. The MyGoals implementation strategies, which are currently being evaluated using the developed evaluation plans, should enable OTs to implement high-quality goal setting and goal management intervention. These efforts to address implementation strategies early and systematically may help bridge the current research-practice gaps in community-based rehabilitation and enhance health in adults with chronic conditions.

## Data Availability Statement

The datasets presented in this article are not readily available because for privacy protection, we do not plan to share the original data. Requests to access the datasets should be directed to EK, eunyoung@wustl.edu.

## Ethics Statement

The studies involving human participants were reviewed and approved by Human Research Protection Office Washington University in St. Louis. The participants provided their consent to participate in this study.

## Author Contributions

EK wrote the manuscript. EF reviewed and edited the manuscript. Both authors contributed to the article and approved the submitted version.

## Funding

This research was funded by the dissertation funding from the Rehabilitation and Participation Science Ph.D. program, Program in Occupational Therapy, Washington University School of Medicine and National Institutes of Health (R21AG063974).

## Conflict of Interest

The authors declare that the research was conducted in the absence of any commercial or financial relationships that could be construed as a potential conflict of interest.

## Publisher's Note

All claims expressed in this article are solely those of the authors and do not necessarily represent those of their affiliated organizations, or those of the publisher, the editors and the reviewers. Any product that may be evaluated in this article, or claim that may be made by its manufacturer, is not guaranteed or endorsed by the publisher.
